# 

**Published:** 1914-12-05

**Authors:** 


### CONTENTS.

PAGE
**Non-Official Hospitals and the British Red Cross Society** ... ... ... ... ... 209
**A Brighton Infirmary for Indian Troops** ... 210
**Hospital and Institutional News** ... ... ... 211
The King in Hospitals in France—Hospital Secretary Killed in Action—England's First Hospital Warrior—Trading with the Enemy?—Three D.S.O.s for "Attending the Wounded"—Bomb-dropping on the Hospital at Bailleul—The Employment of Belgian Pharmacists—The Belgian Language and the Scientific Standard—Twenty-one Years as Treasurer—A Bank Manager's Will—Ireland and the Extension of Medical Benefit—Death of a Hospital Secretary—The Physiology of Soldiers' Songs — *Truth* Christmas Number — The War's Effect on a London Eye Hospital—Increase of Salaries During the War—Mr. Herbert Samuel and Clinical Assistants—The New King George Hospital—Middlesex Hospital and the War—The Women's Voluntary Hospitals Brigade—The Growing List of Notifiable Diseases: Guardians' Criticisms—The Secretary of the "C.O.S." Resigns—Poor-Law Authorities and the War—This Week's Drug Market—To Our Readers.
**Surgical Experiences in the Present War** ... 217
A Fruitful Field of Research.
**How Not to Face a Consultant** ... ... ... 217
**The Mental Deficiency Act** ... ... ... 218
Difficulties of Administration. Much Work—Small Results.
**A Hospital for Soldiers Suffering from Shock** 219
By VISCOUNT KNUTSFORD, Chairman of the London Hospital.

[*See over.*]

### CONTENTS—(*continued*).

PAGE

**Round the World** ... ... ... ... ... ... 220
Fellow-Workers and the Roll of Honour.
**Some Cardinal Points of Voluntary Finance**... 221
The Place of Appeals in Hospital Support.
**Seasonable Appeals in 1914** ... ... ... 222
**The War and the London Hospital** ... ... 223
Review of the Problems at the Quarterly Court.
**The Institutional Library** ... ... ... ... 224
Hahnemann and Modern Medicine.
Wintering Abroad.
A Red Cross Note-Book.
**Leicester Royal Infirmary** ... ... ... .. 225
The Resignation of Sir Edward Wood, J.P.
**Personalia** ... ... ... ... ... ... ... 225
**The Roderick Memorial at Birmingham** ... 226
**Gifts and Bequests** ... ... ... ... ... ... 226
**Buildings Contemplated** ... ... ... ... 226
**Hospitals and the War** ... ... ... ... 227
**Work for the Wounded of the Allied Armies**
Bradford—Bristol—Cardiff : Victims of "General Winter"—Leicester : Arrival of Medical Cases—Norfolk and Norwich Hospital : The Director of Medical Services' Tribute — Tunbridge Wells : A New Problem—Bath : Hospital Visits at 1s. a Head—Walsall : Equipment of Military and Voluntary Hospitals.

PAGE

**Hospital Pharmacists and Olive Oil: Official Tests** ... .. ... ... ... ... ... 228
**The Editor's Letter-Box** ... ... ... ... 229
Vacant Appointments : Should Canvassing be Encouraged or Forbidden?
Discipline in Sanatoria : The Grumbles of Working-class Patients.
A Suggested New Flag for Hospitals.
**The Book Market** ... ... ... ... ... 230
**Institutional Needs** ... ... ... ... ... 230
**The Institutional Worker** ... .. (Suppl.) 1
A General Bulletin Twice Daily.
Daily Bulletins from Wards.
Questions for December.
Questions Invited.
War Matters.
The Sick and in Need.
Employment and Training.

